# Supplementary material for: Antibiotic Prophylaxis or Granulocyte-Colony Stimulating Factor Support in Multiple Myeloma Patients Undergoing Autologous Stem Cell Transplantation
Source: Cancers (Basel). 2021 Jul 9;13(14):3439. doi: 10.3390/cancers13143439 (PMC8303829; doi:10.3390/cancers13143439)
Supplement: Supplementary file 1 [file cancers-13-03439-s001.zip › cancers-1232636-supplementary.pdf]

cancers

MDPI

Table S1. Patients´ characteristics at diagnosis.

Variable	*n* (*n* = 298)	%
Age (*n* = 256)		
Median (range)	58 (51–63) years	
Sex (*n* = 298)		
Female	125	42.0
Male	173	58.0
Heavy chain isotype (*n* = 263)		
IgG	153	58.2
IgA	56	21.3
Bence-Jones	43	16.3
Other	11	4.2
Light chain isotype (*n* = 263)		
Kappa	167	63.5
Lambda	95	36.1
Other	1	0.4
Calcium elevation (*n* = 212)		
Yes	29	13.7
No	183	86.3
Renal insufficiency (*n* = 222)		
Yes	31	14.0
No	191	86.0
Anemia (*n* = 225)		
Yes	75	33.3
No	150	66.7
Bone lesions (*n* = 255)		
Yes	217	85.1
No	38	14.9
ISS (*n* = 165)		
I	77	46.7
II	45	27.3
III	43	26.0
Adverse cytogenetics* (*n* = 188)		
Yes	58	30.9
No	130	69.1

Abbreviations: ISS, International Staging System. * Deletion 17p13, translocation 4; 14 or gain of 1q21 more than three copies.

Table S2. Multivariable mixed effects model on the impact of the anti-infective strategy on the time from platelets < 20/nl to ≥ 20/nl.

Variable	Time to Platelets ≥ 20/nl	
	HR (95% CI)	*p*-value
G-CSF support (vs. antibiotic prophylaxis)	0.86 (0.67–1.09)	0.20
No prophylaxis (vs. antibiotic prophylaxis)	1.12 (0.81–1.55)	0.50
Age (per ten years)	1.00 (0.87–1.16)	0.97
ASCT at relapse (vs. first-line treatment)	0.71 (0.53–0.94)	0.02
≥ VGPR before ASCT (vs. ≤ PR)	0.94 (0.75–1.17)	0.57
Stem cell amount ≥ 2.5* (vs. < 2.5)	1.38 (1.04–1.82)	0.03

Abbreviations: ASCT, autologous stem cell transplantation; CI, confidence interval; G-CSF, granulocyte-colony stimulating factor; HR, Hazard Ratio; PR, partial remission; VGPR, very good partial remission; * ≥ 2.5 × 10^6 CD34+ cells per kg of body weight.

Table S3. Multivariable mixed effects model on the impact of the anti-infective strategy on the total duration of inpatient stay.

**Variable**	**Total duration of inpatient stay**	
	Estimate (95% CI)	*p*-value
G-CSF support (vs. antibiotic prophylaxis)	–0.17 (–0.24—0.10)	<0.001
No prophylaxis (vs. antibiotic prophylaxis)	0.08 (–0.02–0.17)	0.11
Age (per ten years)	0.01 (0.00–0.01)	<0.001
ASCT at relapse (vs. first-line treatment)	0.06 (–0.03–0.14)	0.18
≥ VGPR before ASCT (vs. ≤ PR)	0.05 (–0.02–0.12)	0.15
Stem cell amount ≥ 2.5* (vs. < 2.5)	–0.06 (–0.14–0.02)	0.16

Abbreviations: ASCT, autologous stem cell transplantation; CI, confidence interval; G-CSF, granulocyte-colony stimulating factor; PR, partial remission; VGPR, very good partial remission; * ≥ 2.5 × 10^6 CD34+ cells per kg of body weight.
